# The Influence of Integrated and Intensive Grain Production on the Content and Properties of Chemical Components in Rye Grain

**DOI:** 10.3390/molecules30091880

**Published:** 2025-04-23

**Authors:** Krzysztof Buksa, Alicja Sułek, Michał Szczypek

**Affiliations:** 1 Department of Carbohydrate Technology and Cereal Processing, University of Agriculture in Krakow, Balicka 122, 30-149 Krakow, Poland; 2 Department of Crops and Yield Quality, Institute of Soil Science and Plant Cultivation, State Research Institute, 8 Czartoryskich Street, 24-100 Pulawy, Poland; sulek@iung.pulawy.pl; 3 Faculty of Food Technology, University of Agriculture in Krakow, Balicka 122, 30-149 Krakow, Poland; michal.szczypek@student.urk.edu.pl

**Keywords:** *Secale cereale*, fertilization, polysaccharides, starch, molar mass

## Abstract

The effect of integrated and intensive grain production technologies on the content and properties of chemical components in rye (*Secale cereale* L.) grain of new varieties is not known. This study aimed to examine the effect of production technology on the content and properties of chemical components of rye grain. Grain from four Polish rye varieties obtained as a result of integrated and intensive production was examined. In general, the use of intensive technology resulted in receiving a 7.9% higher yield of grain with a 3.7% higher content of starch, characterized by a higher share of amylose and lower molar mass compared to grain cultivated using the integrated method. Moreover, grain from intensive production contained 0.6% more water-soluble arabinoxylan of a high molar mass but a lower content of ferulic acid, compared to grain obtained by the integrated method. Rye grain from intensive production contained 0.4% more protein, 0.3% more soluble dietary fiber, and similar amounts of phytates than grain cultivated using the integrated method. Regardless of the production method, the hybrid varieties KWS Vinetto and KWS Bono had the highest grain yield and grain with a low content of protein, total and soluble dietary fiber, and extractable arabinoxylan of a high molar mass but low content of ferulic acid.

## 1. Introduction

Rye (*Secale cereale* L.) grain can be produced in an integrated or intensive production system. The integrated agricultural production method is based primarily on protecting the plant from harmful organisms using all available non-chemical methods of plant protection, such as selecting cultivars that are appropriate for the climate and resistant to harmful organisms present in it. Thanks to the use of integrated agricultural production, the risk to human health, animals, and the environment associated with the use of chemical plant protection products in crops can be reduced [1]. Intensive agriculture is a method of farming focused on high efficiency of plant production, which is possible to achieve in highly specialized farms using significant amounts of chemical plant protection products and fertilizers, with relatively low labor inputs. The use of intensive cultivation methods results not only in increased yields but also an increased risk of contamination of soil, water, air, and crops with pesticide residues [2,3].

The use of integrated agricultural production limits the harmful impact of chemical agents used in agrotechnical treatments on the natural environment. Also, it enables the long-term functioning of agricultural economies in a sustainable manner [3,4,5]. However, the use of intensive agricultural production allows for obtaining much larger yields than in the case of cultivation of the same varieties in the integrated agricultural production system [6].

The use of combined fertilization of rye crops with nitrogen, phosphorus, and potassium (NPK) allows for achieving higher yields [7,8,9,10,11,12,13,14].

Nitrogen is necessary for plants to synthesize amino acids, proteins, and nucleic acids. It is also a component of chlorophyll, ADP (adenosine triphosphate), and DNA [15]. Phosphorus is a component of phospholipids, adenosine triphosphate, and nucleic acids [15] and an important component without which some enzymatic reactions cannot occur. Moreover, its deficiency hinders the uptake and assimilation of nitrogen [16,17,18]. Potassium plays a key role in the functioning of the sodium–potassium pump, which is one of the elements maintaining plant homeostasis [19,20].

In the available literature, the effect of NPK fertilization on the chemical composition of rye grain is widely discussed and depends to a large extent on the variety [6,21,22], soil and climatic conditions [23,24,25], the amount, proportion of time and frequency of fertilizer dosing [26,27], production technology [28,29,30,31], and others.

As a result of the studies conducted so far, it has been shown that the use of combined fertilization with NPK generally increases the content of starch and protein in rye grain [32,33]. Moreover, in the case of rye grain as well as other cereals, it was observed that increasing nitrogen fertilization above a certain value, which depends on many factors (type of cereal, variety, and climatic and agrotechnical conditions), results in greater protein biosynthesis and its higher share and, as a consequence of this, a lower share of starch and other components in the mass of the whole grain [33,34,35,36]. Moreover, the rye variety has also been shown to have a large effect on the content of starch, protein, and other grain components [21].

The literature to date lacks information on the effect of fertilization on the dietary fiber content in rye grain. However, studies on cereals other than *Secale cereale* have shown that the effect of NPK fertilization on the dietary fiber content is ambiguous. It has been shown that the use of NPK fertilization generally results in a reduction in the fiber content in the dry matter of the grain [37,38,39,40,41]. According to some reports, the use of fertilization may also result in a lack of effect on the presence of fiber in the grain [42,43].

The effect of fertilization on the share of non-starch polysaccharides in rye grain is also poorly understood. It was only observed that increased fertilization of barley increased the content of beta-glucans in the grain [44].

The effect of fertilization on a higher content of alkylresorcinols in rye grain was found [6,45]. However, there are no reports in the literature on the effect of fertilization on other important components of *Secale cereale* grain, such as phytates. In the studies conducted so far on the effect of fertilization on the chemical composition of wheat grain [35] and millet [46], an increase in the phytate content in the grain was observed.

In Polish conditions, population varieties of rye belonging to the cereal species, as well as hybrid varieties (so-called “hybrids”), are grown. Hybrid varieties are usually characterized by higher yield and grain quality [47,48]. However, there are no reports in the literature on the impact of integrated and intensive production technology on the chemical composition of rye grain.

This study aimed to examine the effect of production technology on the grain yield, the content of chemical components of the greatest importance (such as phytates and dietary fiber), and the molecular properties of starch and arabinoxylans in the grain of rye (*Secale cereale*) of different varieties.

## 2. Results and Discussion

The yield and chemical composition of grain of the examined rye varieties grown in the integrated and intensive production system are presented in Table 1.

According to the statistical analysis (Supplementary Table S3), the yield of winter rye grain depended on the genetic factor and production technology. The highest grain yield was obtained from the hybrid varieties KWS Vinetto and KWS Bono. On average, hybrid *Secale cereale* varieties yielded 8% more compared to population varieties. The highest yield was achieved by the hybrid variety KWS Vinetto (6.9 t/ha). The research by Sułek et al. [49] also indicates that hybrid varieties of winter rye achieve higher grain yields compared to population varieties. According to the results in Table 1 and Supplementary Table S3, a significant effect of production technology on rye grain yield was found. Rye cultivated using intensive technology yielded 7.9% more than that using extensive technology. Grabiński et al. [6] compared the yield of rye at two levels of agricultural technology and observed a positive effect on both hybrid and population varieties produced using intensive technology.

The dry basis (within 88.28–89.86%), starch, and protein contents in rye grain were at typical levels. The starch content in rye grain ranges from 56.0 to 64.0%, with an average of about 60% [21,25,50,51,52]. On the other hand, the protein content in tested rye grain samples is typically in the range of 8 to 13% [50,53]. The content of fatty compounds was also within the range of typical levels of this component in rye grain, determined as 1.8–2.9% [50,53].

The typical ash content in rye grain is assumed to be in the range of 1.7 to 2.0% [50,53,54]. The determined contents of mineral compounds in the tested samples were, in most cases (Table 1), below this range, which could be caused by differences in varieties as well as cultivation conditions.

The total dietary fiber (TDF) content in rye grain typically ranges from 12.6% to 17.08% [28,29,55]. In terms of dietary fiber content, the grain of all rye varieties tested (Table 1) was in the lower range of these typical values. Low ash and fiber content may indicate progress in rye grain breeding because limiting the content of the outer parts of the grain and increasing the share of endosperm in the case of rye grain is desirable. According to the literature data, the typical content of arabinoxylans (AXs) in rye grain is 8–12% [52,54]. The content of total arabinoxylan (Table 1, TAX) in the grain of the rye varieties tested was in this range. Moreover, the TAX content in all samples constituted more than half of the total dietary fiber content, which is consistent with the literature data indicating AX as the most abundant part of rye grain fiber [51,55]. The obtained results showed that the soluble dietary fiber (SDF) fraction consisted almost entirely of water-extractable AX (WEAX, Table 1).

As a result of the conducted analyses (Table 1, the second section; Supplementary Table S3), it was found that the use of intensive production technology resulted in an increase not only in the yield of rye grain but also in the content of starch, protein, total dietary fiber (TDF), soluble dietary fiber fraction (SDF), and water-extractable arabinoxylan (WEAX). However, no significant effect of the applied technology on the content of fat, water-insoluble dietary fiber, and total and water-insoluble AX was found. The observed relationships confirmed the information presented in the literature, according to which increased fertilization results in increased grain yield, mainly through more intensive biosynthesis of starch and protein [28,30,32,33,56]. According to the studies of Menkovska et al. [29] and Łysoń and Biel [28], the production technology also affects the content of TDF. The increased WEAX content observed in rye grain cultivated using intensive technology also corresponds to the literature, which indicates an increase in the content of non-starch polysaccharides (β-glucan) in cereal grain as a result of fertilization [44]. The observed lower ash content in cereal grain cultivated using intensive technology (Table 1) can be explained by a lower share of the outer parts of grain, which is particularly rich in mineral substances in the mass of the whole grain obtained using this production technology [51]. According to the research of Łysoń and Biel [28], organic production technology can result in a higher fat content in rye grain. According to the results presented in Table 1, no significant effect of grain production technology on the fat content in rye grain was observed.

The chemical composition of rye grain also showed significant variation depending on the variety (Table 1, the first section; Supplementary Table S3). The Dańkowskie Granat variety stood out from the others with its average grain yield but had the highest content of protein, ash, and insoluble dietary fiber, including WUAX, along with high starch content at the same time. The obtained results indicate a relatively high share of endosperm in the grain mass but also a relatively thick grain cover. The influence of the variety on the chemical composition of *Secale cereale* grain is documented in the literature [21,24,25,28,49]. The hybrid variety KWS Vinetto was characterized as having the highest yield among the tested varieties and a particularly low content of total dietary fiber. The hybrid variety KWS Bono was also characterized by high yield, high starch content, and low ash content, while the population variety Horyzo was characterized as having the lowest yield and low starch content. No significant effect of the rye variety on the content of fat in the grain was found.

Rye grain may contain significantly higher amounts of inositol phosphates (phytate) than other cereal grains [51]. The properties of inositol phosphates depend on the degree of their phosphorylation. Derivatives with a higher degree of phosphorylation (IP4, IP5, IP6) can form insoluble complexes with multivalent cations, limiting the amount of these elements absorbed from the gastrointestinal tract, and are called mineral absorption inhibitors.

In Table 2, the content of inositol phosphates and total phytate content in the grain of the tested rye varieties grown using an integrated and intensive production technology is presented.

As a result of the conducted studies, it was found that the hexakisphosphate (IP6) form dominated in rye grain and the IP5 content was lower, while the grain of all rye varieties studied contained small amounts of inositol phosphates with a lower degree of phosphorylation. Both the composition and content of phytates in rye grain were typical [57].

The available literature lacks information on the effect of rye grain production technology on the content of inositol phosphates in the grain. As shown in Table 2, both integrated and intensive production technology did not have a significant effect on the content of individual inositol phosphates and total phytate in rye grain. However, the conducted studies showed (Table 2; Supplementary Table S4) that the content of phytate in the grain depended on the rye variety. The population varieties Dańkowskie Granat and Horyzo contained significantly more phytate than the hybrid varieties KWS Vinetto and KWS Bono.

**Figure 1:**
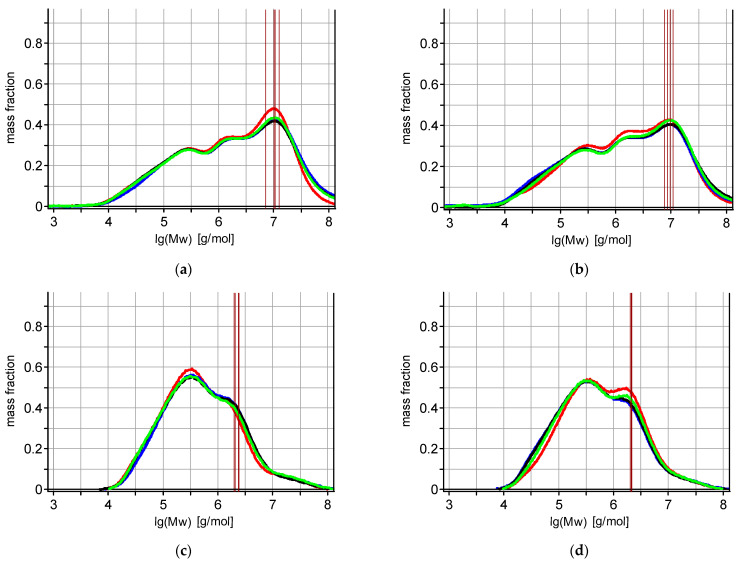
Molecular mass distribution profiles of starches (**a**,**b**) and amylose in starches (**c**,**d**) isolated from the grain of the rye varieties KWS Vinetto (blue), KWS Bono (black), Dańkowskie Granat (green), and Horyzo (red), cultivated according to integrated (**a**,**c**) and intensive (**b**,**d**) methods.

To examine the effect of production technology and variety on the molar mass of starch molecules in rye grain, starch was isolated from wholemeal flour, and then its molecular properties were examined by Size Exclusion Chromatography (SEC). The yield of starch extraction from the grain of the studied rye varieties cultivated using integrated and intensive methods was in the range of 40–60%, and the purity of the obtained starch was above 89.1%. Figure 1 presents molecular mass distribution profiles of starches isolated from the grain of the *Secale cereale* varieties KWS Vinetto, KWS Bono, Dańkowskie Granat, and Horyzo, cultivated according to integrated (Figure 1a) and intensive (Figure 1b) methods.

Starches from rye grain both obtained by integrated and intensive production methods have similar molar mass distribution profiles, where three peaks corresponding to three fractions of low (Mw = 10,000–600,000 g/mol), medium (Mw = 600,000–6,000,000 g/mol) and high (Mw = 6,000,000–100,000,000 g/mol) molar mass were observed (Figure 1).

For detailed information about the molecular structure of amylose in isolated starches, SEC with post-column derivatization was applied. We performed derivatization with iodine, utilizing the known ability of starch fractions to build up complexes detectable at specific wavelengths. The advantage of this novel method is that, after derivatization and detection at 640 nm, general information about the molecular mass distribution of the amylose fraction could be obtained (Figure 1c,d). On the molar mass distribution profiles obtained by SEC with post-column derivatization, two peaks corresponding to two fractions of amylose of low (Mw 10,000–800,000 g/mol) and medium (Mw 800,000–10,000,000 g/mol) molar mass were observed. Moreover, the shape of the molar mass distribution profiles of amylose for samples obtained from rye grain produced by the integrated cultivation method (Figure 1c) was slightly different from the samples obtained by the intensive cultivation method (Figure 1d). On the amylose profiles obtained from rye grain cultivated by the intensive method, two peaks corresponding to two fractions of amylose were more pronounced, compared to samples from the integrated cultivation method.

Based on molecular mass distributions, the molecular parameters of starches and amylose in these starches were calculated. In Table 3, the molecular characteristics of starches isolated from the grain of the studied rye varieties produced by the integrated and intensive methods are shown.

The molar mass of rye starches ranged between 7,550,000 and 11,170,000 g/mol and was similar to the values reported in other studies [51,58].

A significant impact of the technology of grain production on the molar mass of starches was found (Table 3; Supplementary Table S5). Starches isolated from rye grain produced by the integrated method were of higher molar mass than starches from rye grain obtained by the intensive cultivation method. It was also found that starches from the integrated method were less dispersed, so the molecules were of more similar dimensions than those in starches from the intensive method.

The molar mass of starches was also dependent on the rye variety. The highest molar mass of starch was identified in the rye grain of KWS Vinetto and KWS Bono hybrid varieties, whereas the population variety Horyzo contained starch of the lowest molar mass.

To better characterize starches in rye grain, apparent amylose content was determined, and the results were shown in Table 3.

The content of amylose was typical for rye starches and ranged between 22.5 and 24%. A similar range of apparent amylose content was reported in the literature [50,51,52,58]. Starches from the grain of rye cultivated by the intensive method contained higher amounts of amylose. According to the literature, cultivation technology may affect amylose content [59]. In the examined starches, apparent amylose content was not dependent on rye variety (Table 3; Supplementary Table S5).

On the basis of the molecular mass distribution profiles of amylose in isolated starches, the molecular parameters of amylose were calculated and are presented in Table 3.

The molar mass of amylose was significantly lower than the average molar mass of starch [58].

Similarly to whole starch, the molar mass of amylose in starches obtained from rye grain cultivated by integrated technology was significantly higher compared to amylose from rye grain cultivated by the intensive cultivation method; however, amylose in starches from rye grain produced by the intensive method was of lower dispersity, meaning that the amylose molecules were, in this case, more equal in size.

AXs are a very important component of rye grain [5,51]. In order to examine the influence of the cultivation method on AX structure, in the next step water-soluble AXs were extracted from rye grain and examined by SEC chromatography coupled with refractometric and UV detection.

**Figure 2:**
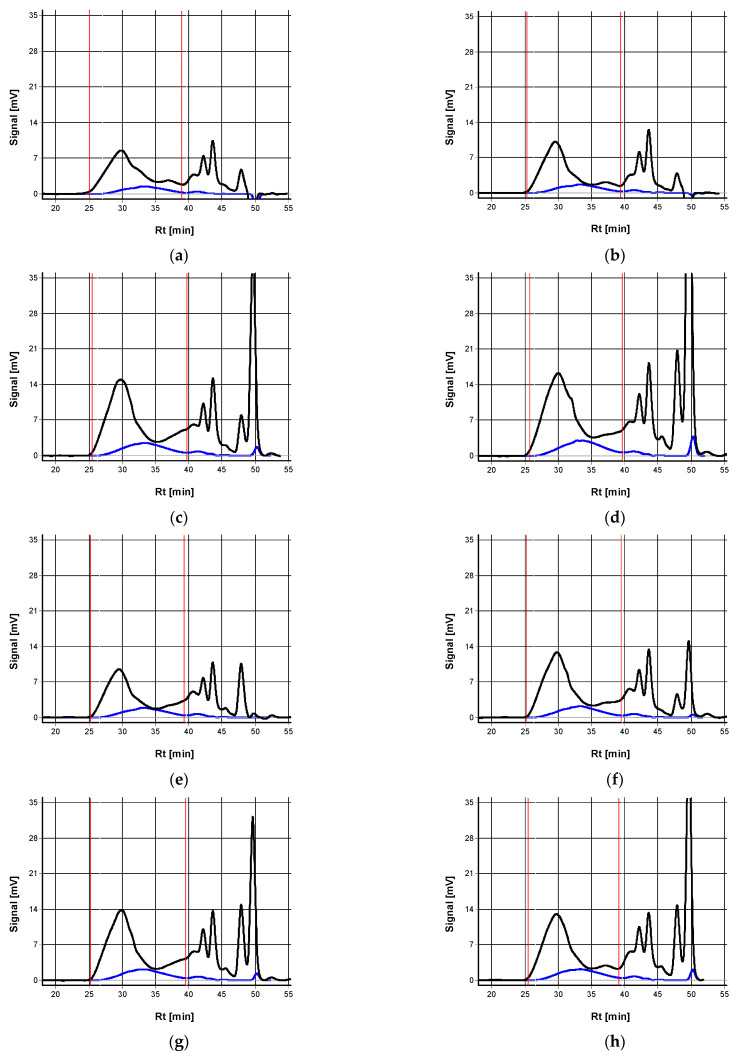
Elution profiles of AX molecules isolated from the grain of the rye varieties KWS Vinetto (**a**,**e**), KWS Bono (**b**,**f**), Dańkowskie Granat (**c**,**g**), and Horyzo (**d**,**h**), cultivated according to integrated (**a**–**d**) and intensive (**e**,**f**) methods, detected by refractometric detector (black line) and ferulic acid distribution within AX molecules (blue line).

In Figure 2, the elution profiles of AX molecules detected by a refractometric detector in rye grain and the ferulic acid distribution within AX molecules are shown. Refractometric detection (Figure 2) allows the detection of AX molecules, whereas UV detection at 320 nm allows the detection of ferulic acid substituting AX molecules and its distribution in AX molecules [60]. According to Figure 2, which shows SEC profiles, three fractions of AX molecules were detected. The highest molar mass fraction was detected between a retention time of 25 to 35 min in all samples. The fractions of medium (retention time between 35 and 45 min) and low (retention time over 45 min) molar mass behave in a less regular manner. In all samples, ferulic acid was detected with the highest content of AX molecules of high and medium molar mass.

Based on the elution profiles, the molecular parameters of AX were calculated, and by comparing the area under the curve recorded by the UV detector at 320 nm with the area of the ferulic acid as the standard, the approximate content of ferulic acid in AX molecules was calculated. In Table 4, the molecular characteristics of arabinoxylans in the grain of the studied rye varieties produced by the integrated and intensive technologies are shown.

Based on the obtained results, it was found that grain obtained as a result of intensive production was characterized by AX with a significantly higher molar mass compared to grain from integrated production (Supplementary Table S5). Grain from intensive production was also characterized by a greater diversity of AX molecules in terms of their size, as evidenced by the greater dispersion of AX molecules (Table 4).

The effect of rye variety on the molecular mass of AX was also observed (Supplementary Table S5). AX of high molar mass was identified in the grain of the hybrid Secale cereale varieties KWS Bono and KWS Vinetto, while the grain of the population variety Horyzo contained AX of the lowest molar mass.

The determined contents of ferulic acid in the extracted AX molecules were similar to those in the literature data [61]. It should be noted that any differences in ferulic acid content could be a consequence of using different methodologies for determining the content of this acid because the SEC/UV method differs from the spectrophotometric and HPLC/UV methods typically used in such determinations.

Based on the results presented in Table 4, a higher content of ferulic acid was found in AX extracted from rye grain cultivated using the intensive method compared to that obtained by the integrated method. Furthermore, it was found that the content of ferulic acid depended on the rye grain variety (Supplementary Table S5). The grain of population varieties, especially the Horyzo variety, contained more of this acid, while the grain of hybrid varieties, especially KWS Vinetto, contained smaller amounts. The dependence of the content of phenolic compounds, including ferulic acid in the grain of the rye variety, is also indicated by the literature data [62].

## 3. Materials and Methods

The research material was the grain of four winter rye (*Secale cereale)* varieties: KWS Bono, KWS Vinetto (hybrid varieties), Horyzo, and Dańkowskie Granat (population varieties) obtained as a result of intensive (A) and integrated (B) production technologies (Supplementary Table S1 and Supplementary Figure S1). Cereal production was carried out in 2019 at the Agricultural Experimental Station in Wielichów (52°07′08″ N 16°20′58″ E), belonging to IUNG-PIB in Puławy. The average temperature in 2019 was 14.00 °C and was higher than the average temperature from 1991 to 2020, which was 11.80 °C. It was a year of rainfall deficit during the rye vegetation period. The total rainfall was 206.8 mm, which was lower than the total rainfall for the multi-year period (512.5 mm). A particularly large rainfall deficit was observed in May and June, the period when plants have the greatest water demand. The experiment was established on good rye complex soil (Brunic Arenosols), in a split-plot design, in three replications. The area of the plots to be harvested was 650 m^2^. Grain of the population rye varieties was sown in the amount of 2.5 million grains/ha and grain of the hybrid rye varieties in the amount of 2.0 million grains/ha. The differences between production technologies are presented in Supplementary Table S2.

### 3.1. Grain Milling and Analysis

Rye grain was milled and sieved through a 0.5 mm sieve using a laboratory mill Foss Tecator.

Dry basis content was determined according to Method 925.10 [63].

Starch content was determined using the enzymatic HPLC method according to Buksa [58]. To determine starch content, 100 mg of flour was weighed into test tubes. Then, 10 mL of sodium acetate buffer (pH = 5, 100 mM) containing calcium chloride (5 mM) was added. The sample was mixed vigorously. To the prepared sample, 0.1 mL of thermostable α-amylase (*Bacillus licheniformis*; 3000 U/mL; Sigma-Aldrich, Saint Louis, MO, USA) was added. The sample was mixed using a vortex. The prepared sample was placed in a hot water bath. Fifteen minutes after adding the enzyme, the sample was removed from the water bath and mixed vigorously using a vortex. The sample was then cooled in a water bath at 50 °C, and 0.1 mL of undiluted amyloglucosidase enzyme (Megazyme, Bray, Ireland; 3300 U/mL) was added to the sample, mixed using a vortex, and then incubated for 30 min at 50 °C. After incubation, 0.1 mL of the sample solution was placed in an Eppendorf tube, 0.9 mL of distilled water was added, the diluted sample was centrifuged (21,000× *g*; 10 min), and 20 µL of the supernatant was injected into the HPLC/RI system. Chromatographic analysis was performed at 60 °C; the mobile phase was 0.005 M H_2_SO_4_. The starch content was calculated based on the determined glucose content multiplied by a polymerization coefficient of 0.9.

The protein content was determined according to Kjeldahl’s method (using a conversion factor of 6.25), according to Method 950.36 [63].

Fat content was determined according to Method 922.06 [63] 

Ash content was determined according to Method 930.05 [63].

The determination of soluble (SDF), insoluble (IDF), and total dietary fiber (TDF) was performed according to Method 991.43 [63] using the Total Dietary Fiber Kit (Megazyme, Bray, Ireland).

Total (TAX), water-extractable (WEAX), and water-unextractable (WUAX) arabinoxylans were determined according to Hashimoto, Shogren, and Pomeranz [64].

Total phytate and inositol phosphate determination was performed according to [65,66]. The extraction of inositol phosphates from samples was conducted according to Sandberg et al. [66]. Briefly, 0.5 g of the samples was extracted in 20 mL of 0.5 M hydrochloric acid at 20 °C for 3 h. Extracts were centrifuged for 10 min at 6000× *g*, frozen overnight, centrifuged again, and separated from the extract by ion exchange chromatography using 2 g of AG 1 × X8 anion exchanger (200–400-mesh, Bio-Rad; Hercules, CA, USA). Eluates were evaporated and redissolved in deionized water and injected into the HPLC/RI system (Knauer, Germany). The separation of inositol phosphates was performed using an Aquasil C18 column (with guard column) at 40 °C and a flow rate of 0.8 mL/min. The mobile phase consisted of 0,05 M formic acid:methanol (HPLC grade) 49:51, and 1.5 mL/100 mL of tertbutylammonium hydroxide (40%, Sigma-Aldrich, Saint Louis, MO, USA) was added. The pH was adjusted to 4.3 by the addition of sulfuric acid. The calibration of the HPLC/RI system was performed using sodium phytate (Sigma-Aldrich, Saint Louis, MO, USA) as the external standard, and standard solutions containing 0.05–1.0 µmol/mL of phytate were prepared. Correction factors for differences in the detector response of inositol hexa-, penta-, tetra-, and triphosphates were calculated as described previously [65,66].

### 3.2. Extraction of Rye Starch and Its Analysis

The extraction of rye starch was performed using the xylanase–protease-based enzymatic method by Buksa [58]. In short, rye grain was milled into flour using a Quadrumat Junior (Labor Muszeripari Muwek type QG-109, Hungary) mill. A total of 50 g (dry basis) of rye flour was suspended in 175 mL of 50 mM MES buffer (pH adjusted to 6.0 with 6 M NaOH) containing 0.005% sodium azide. A total of 66 U of xylanase from *Thermomyces lanuginosus* (Sigma-Aldrich, Saint Louis, MO, USA) was added, and the suspension was incubated with continuous stirring for 20 h at 40 °C. After incubation, 175 mL of 50 mM TRIS buffer was added to the suspension, and the pH was adjusted with 6 M NaOH to 8.2; then, 55 U of protease from *Streptomyces griseus* (Sigma-Aldrich, Saint Louis, MO, USA) was added, and the suspension was further incubated with continuous stirring at 8 °C for 48 h. After complete incubation with xylanase and protease, the digested material was passed through a 1.0 mm sieve and flushed 3 times with 20 mL of water, and the obtained filtrate was centrifuged at 2000× *g* for 10 min. The supernatant was discarded, and the sediment was resolved in 175 mL of water and centrifuged again (10 min; 2000× *g*). The supernatant was discarded, and from the sediment, a clear layer of starch was manually separated. The rest of the crude starch was resolved in 27.5 mL of 50 mM MES buffer, 66 U of xylanase was added, and the suspension was incubated with continuous stirring for 10 h at 40 °C; this was followed by the addition of 27.5 mL of 50 mM TRIS (pH adjusted to 8.2) and 55 U of protease and incubation with continuous stirring for 48 h at 8 °C. After complete incubation, the suspension was centrifuged at 2000× *g* for 10 min, the supernatant was discarded, a thin grayish layer was scraped off from the starch surface, and the residue was resolved in 175 mL of water and centrifuged (10 min; 2000× *g*). This step was repeated until the starch was clean in appearance. The resulting starch obtained during isolation was combined. Finally, the starches were dried at room temperature and sieved through a 0.12 mm sieve using a mortar and pestle.

Yields of starch are expressed as a percentage of the total starch content of flour.

The determination of the molecular properties of the isolated starches was performed according to Buksa [58], by HPSEC/RI with post-column derivatization and UV/VIS detection. In short, 20 mg of the sample was dissolved in 6 mL DMSO at 100 °C for 24 h. The solution was centrifuged, and 100 μL of the supernatant was injected into the HPSEC system consisting of OHpak columns SB-806 and SB-804 (Shodex, Japan) operating at 60 °C. An aqueous solution of 100 mM NaNO_3_ was used as an eluent at a flow rate of 0.6 mL/min. The molecular mass distribution of the starch was measured using refractometric (RI) detection. For post-column derivatization, the output of the last HPSEC column was connected to the post-column mixer, reaction coil (200 μL capacity), and the UV/VIS detector operating at 640 nm. The temperature of the post-column mixer and reaction coil was maintained at 60 °C. A freshly prepared solution of 10% I_2_-DMSO (90% DMSO, 10% water, 0.006M I_2_), 10% 6 M urea, and 80% water was used as a post-column derivatization reagent at a flow rate of 0.4 mL/min.

Calibration of the HPSEC systems was performed with pullulan standards (Shodex Standard, Macherey–Nagel) with known molecular masses, maltose, and glucose. Molecular mass distributions were used to calculate the apparent molecular parameters: average molar mass (Mw, Mn) and the dispersity (Ð = Mw/Mn) using Eurochrom (ver. 3.05, Knauer, Berlin, Germany) and Clarity (ver. 4.0.1.700, DataApex, Prague, Czech Republic) software.

Apparent amylose content was determined according to Morrison and Laignelet [67].

### 3.3. Extraction of Rye Arabinoxylan and Its Analysis

The extraction and purification of extractable arabinoxylan were performed according to Shewry [68].

The distribution of the molar mass of AX was evaluated by HPSEC/RI/UV. A total of 100 μL of AX dissolved in 100 mM NaNO_3_ was injected into the HPSEC system equipped with a combination of OHpak SB-G, SB-806HQ, SB-804HQ, and SB-802HQ (Shodex, Japan) columns. The samples were detected with RI and UV (at 320 nm, for ferulic acid detection, Buksa [60]) detectors. As an SEC eluent, 100 mM NaNO_3_ was used at a flow rate of 0.6 mL/min. Separation was performed at a column temperature of 60 °C. The calibration of the SEC system was performed with pullulan standards and arabinose (Sigma-Aldrich, Saint Louis, MO, USA). The molecular mass distribution and apparent average molar mass Mw (related to pullulans), as well as ferulic acid content (related to ferulic acid standard), were calculated with the software programs Eurochrom ver. 3.05 (Knauer, Berlin, Germany) and Clarity ver. 4.0.1.700 (DataApex, Prague, Czech Republic).

### 3.4. Statistical Evaluation of Results

All analyses were performed at least in triplicate, and the obtained results were subjected to a two-factor analysis of variance (ANOVA) using the StatSoft Statistica v. 9.0 (StatSoft, Inc., Tulsa, OK, USA), differentiating the samples depending on the grain variety and the technology of cultivation. The significance of differences between the average values was verified by Tukey’s test at a significance level of 0.05.

## 4. Conclusions

In summary, the use of intensive technology resulted in obtaining rye grain with a 3.7% higher content of starch, 0.4% more protein, 0.3% more soluble dietary fiber, 0.6% more WEAX, and similar contents of phytates, compared to grain cultivated using the integrated method. Starch in rye grains produced by intensive technology was characterized by a higher (of 0.9%) share of amylose and a 9.6% lower molar mass, compared to starches in grain cultivated using the integrated method. WEAX in rye grain produced by intensive technology were of 2.9% higher molar mass and generally contained 21.2% less ferulic acid in the AX molecules, compared to WEAX from *Secale cereale* grain cultivated using the integrated method. Regardless of the method of grain production, the grain of the Horyzo variety had the lowest grain yield and the lowest content of starch, characterized by a low molar mass, high content of phytates, and a high content of WEAX of a low molar mass but a high degree of substitution with ferulic acid. Regardless of the method of grain production, the hybrid varieties KWS Vinetto and KWS Bono had the highest grain yield and grain with a low content of protein, total dietary fiber, and soluble dietary fiber, including WEAX of a high molar mass and low content of ferulic acid.

## Figures and Tables

**Table 1:** Yield of grain and chemical composition of rye grain.

		Tech *	Yield [t/ha]	Starch [%]	Protein [%]	Fat [%]	Ash [%]	TDF ** [%]	SDF ** [%]	IDF ** [%]	WEAX [%]	WUAX [%]	TAX [%]
Variety	KWS Vinetto		6.9 ± 0.2 ^d^	63.3 ± 6.0 ^b^	10.7 ± 0.3 ^a^	2.2 ± 0.1 ^a^	1.57 ± 0.05 ^b^	14.4 ± 0.3 ^a^	3.4 ± 0.1 ^a^	11.0 ± 0.2 ^b^	3.1 ± 0.4 ^a^	5.4 ± 0.7 ^a^	8.5 ± 0.9 ^a^
Variety	KWS Bono		6.7 ± 0.6 ^bc^	64.5 ± 0.8 ^c^	10.8 ± 0.2 ^b^	2.3 ± 0.1 ^a^	1.54 ± 0.02 ^a^	14.9 ± 0.8 ^c^	3.4 ± 0.1 ^a^	11.5 ± 0.8 ^c^	3.1 ± 0.3 ^a^	5.3 ± 0.5 ^a^	8.4 ± 0.3 ^a^
Variety	D. Granat		6.6 ± 0.2 ^b^	65.1 ± 0.2 ^c^	11.9 ± 0.0 ^d^	2.4 ± 0.1 ^a^	1.61 ± 0.02 ^c^	15.9 ± 0.3 ^d^	3.9 ± 0.1 ^b^	12.0 ± 0.2 ^d^	3.7 ± 0.1 ^b^	6.6 ± 0.6 ^b^	10.4 ± 0.5 ^b^
Variety	Horyzo		6.0 ± 0.2 ^a^	61.2 ± 1.9 ^a^	11.8 ± 0.4 ^c^	2.3 ± 0.2 ^a^	1.57 ± 0.23 ^b^	14.6 ± 0.1 ^b^	3.9 ± 0.6 ^b^	10.7 ± 0.5 ^a^	3.9 ± 0.6 ^b^	5.3 ± 0.6 ^a^	9.2 ± 0.4 ^a^
Tech	ID		6.3 ± 0.4 ^a^	61.7 ± 3.1 ^a^	11.1 ± 0.7 ^a^	2.3 ± 0.1 ^a^	1.61 ± 0.11 ^b^	14.7 ± 0.8 ^a^	3.5 ± 0.3 ^a^	11.3 ± 0.6 ^a^	3.1 ± 0.4 ^a^	5.8 ± 0.9 ^a^	9.0 ± 1.2 ^a^
Tech	IE		6.8 ± 0.5 ^b^	65.4 ± 2.1 ^b^	11.5 ± 0.6 ^b^	2.3 ± 0.1 ^a^	1.54 ± 0.10 ^a^	15.1 ± 0.5 ^b^	3.8 ± 0.4 ^b^	11.3 ± 0.8 ^a^	3.7 ± 0.4 ^b^	5.5 ± 0.6 ^a^	9.2 ± 0.6 ^a^
Var × Tech ***	KWS Vinetto	ID	6.9 ± 0.2 ^cd^	58.2 ± 0.2 ^a^	10.4 ± 0.1 ^a^	2.2 ± 0.1 ^a^	1.53 ± 0.01 ^b^	14.2 ± 0.1 ^a^	3.3 ± 0.0 ^a^	10.9 ± 0.1 ^bc^	2.8 ± 0.0 ^a^	4.9 ± 0.4 ^a^	7.7 ± 0.4 ^a^
Var × Tech	KWS Vinetto	IE	7.0 ± 0.1 ^cd^	68.5 ± 0.2 ^e^	10.9 ± 0.0 ^b^	2.3 ± 0.1 ^a^	1.62 ± 0.01 ^c^	14.6 ± 0.0 ^b^	3.4 ± 0.0 ^a^	11.1 ± 0.0 ^bc^	3.4 ± 0.1 ^bc^	5.8 ± 0.7 ^ab^	9.2 ± 0.6 ^abc^
Var × Tech	KWS Bono	ID	6.2 ± 0.1 ^ab^	63.8 ± 0.3 ^c^	10.7 ± 0.0 ^a^	2.2 ± 0.1 ^a^	1.53 ± 0.00 ^b^	14.2 ± 0.1 ^a^	3.4 ± 0.1 ^a^	10.8 ± 0.1 ^b^	2.8 ± 0.0 ^a^	5.5 ± 0.4 ^ab^	8.4 ± 0.4 ^ab^
Var × Tech	KWS Bono	IE	7.3 ± 0.0 ^d^	65.2 ± 0.3 ^d^	11.0 ± 0.0 ^b^	2.3 ± 0.0 ^a^	1.56 ± 0.01 ^b^	15.5 ± 0.0 ^c^	3.4 ± 0.1 ^a^	12.2 ± 0.1 ^e^	3.3 ± 0.1 ^b^	5.1 ± 0.5 ^a^	8.4 ± 0.4 ^ab^
Var × Tech	D. Granat	ID	6.5 ± 0.3 ^bc^	65.2 ± 0.3 ^d^	12.0 ± 0.0 ^d^	2.4 ± 0.1 ^a^	1.63 ± 0.01 ^c^	16.1 ± 0.1 ^d^	3.9 ± 0.1 ^b^	12.2 ± 0.0 ^e^	3.7 ± 0.1 ^cd^	7.1 ± 0.1 ^b^	10.8 ± 0.1 ^c^
Var × Tech	D. Granat	IE	6.7 ± 0.1 ^bc^	65.0 ± 0.1 ^d^	11.9 ± 0.0 ^d^	2.4 ± 0.0 ^a^	1.60 ± 0.01 ^c^	15.6 ± 0.0 ^c^	3.9 ± 0.1 ^b^	11.8 ± 0.0 ^d^	3.8 ± 0.0 ^d^	6.0 ± 0.2 ^ab^	9.9 ± 0.2 ^bc^
Var × Tech	Horyzo	ID	5.8 ± 0.1 ^a^	59.6 ± 0.2 ^b^	11.5 ± 0.1 ^c^	2.2 ± 0.1 ^a^	1.77 ± 0.01 ^d^	14.5 ± 0.0 ^b^	3.3 ± 0.1 ^a^	11.2 ± 0.1 ^c^	3.3 ± 0.1 ^bc^	5.8 ± 0.4 ^ab^	9.2 ± 0.4 ^abc^
Var × Tech	Horyzo	IE	6.2 ± 0.1 ^ab^	62.9 ± 0.2 ^c^	12.1 ± 0.0 ^d^	2.4 ± 0.2 ^a^	1.38 ± 0.01 ^a^	14.7 ± 0.1 ^b^	4.4 ± 0.0 ^c^	10.3 ± 0.1 ^a^	4.3 ± 0.1 ^e^	4.9 ± 0.4 ^a^	9.2 ± 0.4 ^abc^

* Tech—technology: ID—integrated, IE—intensive; ** TDF—total dietary fiber, SDF—soluble dietary fiber, IDF—insoluble dietary fiber. WEAX, WUAX, and TAX—water-extractable, water-insoluble, and total arabinoxylan content, respectively. *** The interaction of variety and technology. Mean values marked with the same letters in particular columns are not statistically significantly different at *p* < 0.05.

**Table 2:** Content of inositol phosphates and total phytate in rye grain.

		Tech *	IP3 ** [%]	IP4 ** [%]	IP5 ** [%]	IP6 ** [%]	Total Phytate [%]
Variety	KWS Vinetto		0.004 ± 0.002 ^a^	0.007 ± 0.002 ^a^	0.066 ± 0.006 ^a^	1.720 ± 0.051 ^a^	1.797 ± 0.057 ^a^
Variety	KWS Bono		0.005 ± 0.003 ^a^	0.006 ± 0.002 ^a^	0.059 ± 0.011 ^a^	1.686 ± 0.137 ^a^	1.755 ± 0.145 ^a^
Variety	D. Granat		0.004 ± 0.003 ^a^	0.006 ± 0.002 ^a^	0.069 ± 0.009 ^a^	1.826 ± 0.273 ^ab^	1.905 ± 0.280 ^ab^
Variety	Horyzo		0.005 ± 0.004 ^a^	0.006 ± 0.001 ^a^	0.066 ± 0.010 ^a^	1.946 ± 0.114 ^b^	2.022 ± 0.119 ^b^
Tech	ID		0.003 ± 0.002 ^a^	0.007 ± 0.002 ^a^	0.067 ± 0.005 ^a^	1.792 ± 0.175 ^a^	1.869 ± 0.178 ^a^
Tech	IE		0.005 ± 0.004 ^a^	0.006 ± 0.001 ^a^	0.063 ± 0.012 ^a^	1.796 ± 0.199 ^a^	1.870 ± 0.207 ^a^
Var × Tech ***	KWS Vinetto	ID	0.002 ± 0.001 ^a^	0.008 ± 0.001 ^a^	0.069 ± 0.003 ^a^	1.748 ± 0.015 ^a^	1.828 ± 0.017 ^a^
Var × Tech	KWS Vinetto	IE	0.005 ± 0.000 ^a^	0.005 ± 0.001 ^a^	0.063 ± 0.008 ^a^	1.692 ± 0.067 ^a^	1.766 ± 0.075 ^a^
Var × Tech	KWS Bono	ID	0.002 ± 0.001 ^a^	0.008 ± 0.001 ^a^	0.066 ± 0.003 ^a^	1.787 ± 0.077 ^ab^	1.862 ± 0.077 ^ab^
Var × Tech	KWS Bono	IE	0.007 ± 0.003 ^a^	0.005 ± 0.001 ^a^	0.051 ± 0.010 ^a^	1.585 ± 0.097 ^a^	1.649 ± 0.105 ^a^
Var × Tech	D. Granat	ID	0.006 ± 0.001 ^a^	0.006 ± 0.001 ^a^	0.062 ± 0.007 ^a^	1.597 ± 0.051 ^a^	1.670 ± 0.057 ^a^
Var × Tech	D. Granat	IE	0.002 ± 0.002 ^a^	0.007 ± 0.003 ^a^	0.076 ± 0.001 ^a^	2.055 ± 0.104 ^b^	2.140 ± 0.106 ^b^
Var × Tech	Horyzo	ID	0.002 ± 0.000 ^a^	0.006 ± 0.002 ^a^	0.071 ± 0.001 ^a^	2.038 ± 0.067 ^b^	2.118 ± 0.068 ^b^
Var × Tech	Horyzo	IE	0.007 ± 0.006 ^a^	0.005 ± 0.001 ^a^	0.060 ± 0.014 ^a^	1.853 ± 0.018 ^ab^	1.926 ± 0.027 ^ab^

* Tech—technology: ID—integrated, IE—intensive; ** IP3, IP4, IP5, and IP6 inositol phosphates; *** the interaction of variety and technology. Mean values marked with the same letters in particular columns are not statistically significantly different at *p* < 0.05.

**Table 3:** Molecular characteristics of starches and amylose in starches isolated from rye grain.

		Tech *	Mw × 10^6^ [g/mol]	Mn × 10^6^ [g/mol]	Ð	Amylose Mw × 10^6^ [g/mol]	Amylose Mn × 10^6^ [g/mol]	Amylose Ð	Apparent Amylose Content [%]
Variety	KWS Vin.		10.76 ± 2.27 ^bc^	0.12 ± 0.09 ^a^	124.3 ± 68.6 ^d^	2.366 ± 0.151 ^b^	0.180 ± 0.017 ^ab^	13.2 ± 0.4 ^d^	22.9 ± 0.47 ^a^
Variety	KWS Bono		11.17 ± 0.37 ^c^	0.16 ± 0.02 ^c^	70.9 ± 6.0 ^b^	2.072 ± 0.048 ^a^	0.176 ± 0.008 ^a^	11.8 ± 0.3 ^b^	23.3 ± 0.75 ^a^
Variety	D. Granat		10.15 ± 0.39 ^b^	0.13 ± 0.03 ^a^	84.0 ± 17.3 ^c^	2.306 ± 0.193 ^b^	0.183 ± 0.006 ^ab^	12.6 ± 1.3 ^c^	23.4 ± 0.56 ^a^
Variety	Horyzo		7.55 ± 0.25 ^a^	0.15 ± 0.01 ^b^	51.6 ± 4.1 ^a^	2.113 ± 0.086 ^a^	0.194 ± 0.025 ^b^	11.0 ± 1.0 ^a^	23.0 ± 0.44 ^a^
Tech	ID		10.41 ± 2.06 ^b^	0.16 ± 0.02 ^b^	64.5 ± 11.0 ^a^	2.272 ± 0.222 ^b^	0.181 ± 0.010 ^a^	12.5 ± 0.9 ^b^	22.7 ± 0.24 ^a^
Tech	IE		9.41 ± 1.45 ^a^	0.11 ± 0.05 ^a^	100.9 ± 54.0 ^b^	2.156 ± 0.092 ^a^	0.185 ± 0.021 ^a^	11.8 ± 1.3 ^a^	23.6 ± 0.37 ^b^
Var × Tech ***	KWS Vin.	ID	12.71 ± 0.35 ^d^	0.20 ± 0.00 ^e^	64.8 ± 0.4 ^c^	2.480 ± 0.086 ^c^	0.194 ± 0.005 ^bc^	12.8 ± 0.1 ^c^	22.5 ± 0.32 ^a^
Var × Tech	KWS Vin.	IE	8.80 ± 0.36 ^ab^	0.05 ± 0.00 ^a^	183.7 ± 0.0 ^g^	2.252 ± 0.093 ^abc^	0.166 ± 0.007 ^a^	13.6 ± 0.0 ^d^	23.3 ± 0.16 ^abc^
Var × Tech	KWS Bono	ID	11.07 ± 0.46 ^c^	0.15 ± 0.01 ^c^	76.1 ± 0.0 ^e^	2.092 ± 0.058 ^a^	0.181 ± 0.007 ^ab^	11.6 ± 0.2 ^b^	22.7 ± 0.16 ^a^
Var × Tech	KWS Bono	IE	11.27 ± 0.39 ^c^	0.17 ± 0.00 ^d^	65.8 ± 0.9 ^cd^	2.051 ± 0.043 ^a^	0.171 ± 0.007 ^ab^	12.0 ± 0.2 ^b^	24.0 ± 0.34 ^c^
Var × Tech	D. Granat	ID	10.40 ± 0.43 ^c^	0.15 ± 0.00 ^c^	69.1 ± 0.9 ^d^	2.461 ± 0.101 ^bc^	0.178 ± 0.005 ^ab^	13.8 ± 0.2 ^d^	22.9 ± 0.32 ^ab^
Var × Tech	D. Granat	IE	9.91 ± 0.21 ^bc^	0.10 ± 0.00 ^b^	98.9 ± 2.0 ^f^	2.151 ± 0.074 ^a^	0.187 ± 0.004 ^ab^	11.5 ± 0.2 ^b^	23.8 ± 0.17 ^bc^
Var × Tech	Horyzo	ID	7.44 ± 0.31 ^a^	0.15 ± 0.01 ^cd^	48.1 ± 0.3 ^a^	2.056 ± 0.085 ^a^	0.173 ± 0.007 ^ab^	11.9 ± 0.0 ^b^	22.7 ± 0.16 ^a^
Var × Tech	Horyzo	IE	7.67 ± 0.21 ^a^	0.14 ± 0.01 ^c^	55.1 ± 0.7 ^b^	2.170 ± 0.045 ^ab^	0.214 ± 0.009 ^c^	10.1 ± 0.2 ^a^	23.4 ± 0.33 ^abc^

* Tech—technology: ID—integrated, IE—intensive; Mw—weight average molar mass, Mn—number average molar mass, Ð—dispersity. *** The interaction of variety and technology. Mean values marked with the same letters in particular columns are not statistically significantly different at *p* < 0.05.

**Table 4:** Molecular characteristics of water-extractable arabinoxylan (AX) in rye grain and ferulic acid (FA) content in AX molecules.

		Tech *	AX Mw × 10^6^ [g/mol]	AX Mn × 10^6^ [g/mol]	AX Ð	FA ** Content [mg/100g AX]
Variety	KWS Vin.		2.066 ± 0.031 ^c^	0.149 ± 0.023 ^b^	14.1 ± 2.4 ^c^	2.6 ± 0.7 ^a^
Variety	KWS Bono		2.065 ± 0.112 ^c^	0.159 ± 0.029 ^d^	13.2 ± 1.7 ^b^	2.9 ± 0.7 ^b^
Variety	D. Granat		1.943 ± 0.053 ^b^	0.113 ± 0.009 ^a^	17.2 ± 0.9 ^d^	4.3 ± 0.2 ^c^
Variety	Horyzo		1.871 ± 0.159 ^a^	0.152 ± 0.040 ^c^	12.8 ± 2.3 ^a^	4.7 ± 0.3 ^d^
Tech	ID		1.958 ± 0.171 ^a^	0.144 ± 0.036 ^a^	14.1 ± 2.7 ^a^	3.3 ± 1.2 ^a^
Tech	IE		2.015 ± 0.051 ^b^	0.143 ± 0.028 ^a^	14.5 ± 2.4 ^b^	4.0 ± 0.7 ^b^
Var × Tech ***	KWS Vin.	ID	2.039 ± 0.001 ^e^	0.169 ± 0.001 ^f^	12.0 ± 0.0 ^b^	2.0 ± 0.0 ^a^
Var × Tech	KWS Vin.	IE	2.093 ± 0.001 ^f^	0.129 ± 0.001 ^d^	16.2 ± 0.2 ^d^	3.2 ± 0.1 ^b^
Var × Tech	KWS Bono	ID	2.162 ± 0.016 ^g^	0.184 ± 0.001 ^g^	11.7 ± 0.0 ^b^	2.4 ± 0.0 ^a^
Var × Tech	KWS Bono	IE	1.969 ± 0.008 ^c^	0.135 ± 0.002 ^e^	14.6 ± 0.1 ^c^	3.5 ± 0.1 ^b^
Var × Tech	D. Granat	ID	1.897 ± 0.001 ^b^	0.105 ± 0.001 ^a^	18.0 ± 0.2 ^e^	4.4 ± 0.0 ^c^
Var × Tech	D. Granat	IE	1.989 ± 0.001 ^cd^	0.121 ± 0.001 ^c^	16.5 ± 0.1 ^d^	4.2 ± 0.2 ^c^
Var × Tech	Horyzo	ID	1.733 ± 0.004 ^a^	0.117 ± 0.002 ^b^	14.8 ± 0.2 ^c^	4.5 ± 0.0 ^cd^
Var × Tech	Horyzo	IE	2.009 ± 0.007 ^d^	0.187 ± 0.000 ^g^	10.7 ± 0.0 ^a^	5.0 ± 0.3 ^d^

* Tech—technology: ID—integrated, IE—intensive; ** FA—ferulic acid; Mw—weight average molar mass, Mn—number average molar mass, Ð—dispersity. *** The interaction of variety and technology. Mean values marked with the same letters in particular columns are not statistically significantly different at *p* < 0.05.

## Data Availability

Data are contained within this article and Supplementary Materials.

## Supplementary Materials

The following supporting information can be downloaded at https://www.mdpi.com/article/10.3390/molecules30091880/s1, Table S1. Characteristics of rye grain. Table S2. Characterization of applied technologies for rye winter production. Table S3. Analysis of variance (two-way ANOVA). The effect of variety and technology of grain production on composition of rye grain. Table S4. Analysis of variance (two-way ANOVA). The effect of variety and technology of grain production on the content of phytates. Table S5. Analysis of variance (two-way ANOVA). The effect of variety and technology of grain production on molecular properties of starch and water soluble arabinoxylan (WEAX). Figure S1. Grain of rye varieties. (A) KWS Vinetto, (B) KWS Bono, (C) Dańkowskie Granat and (D) Horyzo.
