# Supplementary material for: The Influence of Integrated and Intensive Grain Production on the Content and Properties of Chemical Components in Rye Grain
Source: Molecules. 2025 Apr 23;30(9):1880. doi: 10.3390/molecules30091880 (PMC12073726; doi:10.3390/molecules30091880)
Supplement: Supplementary file 1 [file molecules-30-01880-s001.zip › molecules-3560311-supplementary.pdf]

Supplementary Table S1. Characteristics of rye grain.

	Thousand kernel weight (g)		Test weight (kg·hL^{-1})	
Production technology	Integrated	Intensive	Integrated	Intensive
Variety of rye				
KWS Vinetto	23,90	27,69	72,2	73,2
KWS Bono	29,32	28,62	71,7	72,1
Dańkowskie Granat	27,28	26,54	72,8	73,8
Horyzo	33,74	26,22	73,8	74,0

Supplementary Table S2. Characterization of applied technologies for rye winter production.

**Specification**	**Production technology**	
	**Integrated**	**Intensive**
Fertilization (kg·ha^{-1})		
N (ammonium nitrate)	50 (start of vegetation)	50 (start of vegetation)
	30 (at BBCH 51)	50 (at BBCH 31)
		20 (at BBCH 51)
P (superphosphate)	40	60
K (potassium salt)	60	90
Herbicide (g·ha^{-1})	At BBCH 20<br>Snajper 600 SC (difufenican+ chlorotoluron) (1,0)<br>Lentipur Flo 500 SC (chlorotoluron) (1,0)	
Fungicide (L·ha^{-1})	Not applied	At BBCH 31<br>Fossa 633 EC (phenopropidine + prohlorase)<br>(1,2)<br>Kosa 250 EW (tebuconazole)<br>(1,0)
Growth regulator (L·ha^{-1})	At BBCH 39<br>Stiff 250 SC (trinexapac-ethyl)<br>(0,3)	Not applied

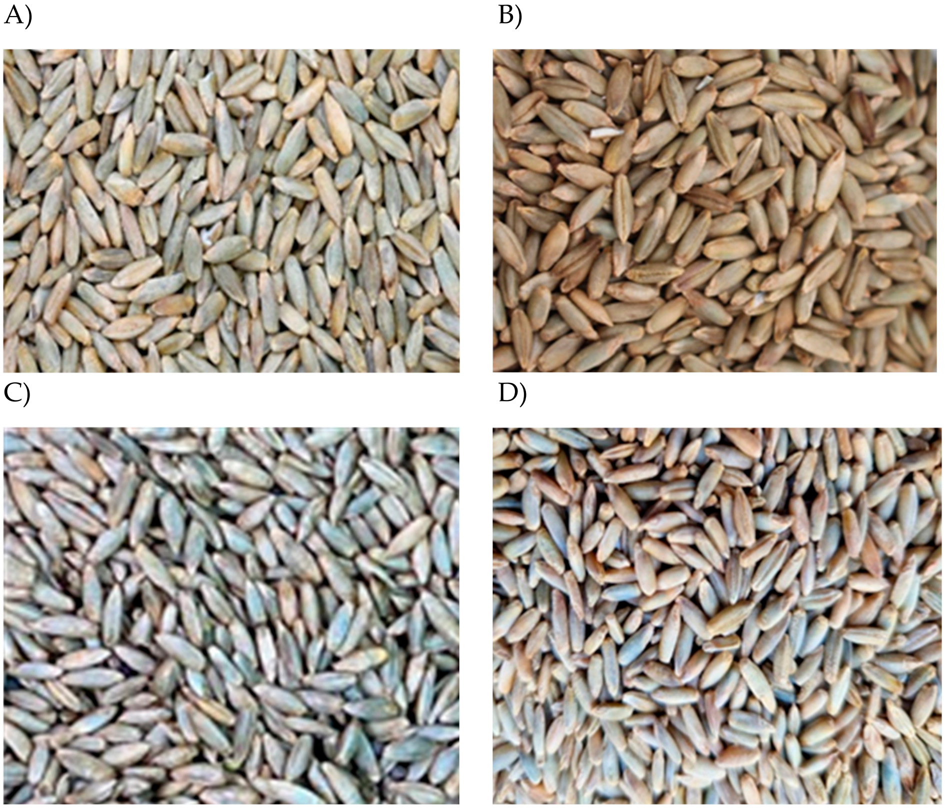

Supplementary Figure S1. Grain of rye varieties. A) KWS Vinetto, B) KWS Bono, C) Dańkowskie Granat and D) Horyzo.

Supplementary Table S3. Analysis of variance (two-way ANOVA). The effect of variety and technology of grain production on composition of rye grain.

	Yield		Starch		Protein		Fat		Ash		TDF		SDF		IDF		WEAX		WUAX		TAX	
	F	p	F	p	F	p	F	p	F	p	F	p	F	p	F	p	F	p	F	p	F	p
Variety	31,8	*	175,6	*	497,2	*	1,5	ns	49,2	*	326,9	*	67,0	*	151,0	*	98,6	*	8,0	*	20,0	*
Technology	40,1	*	834,7	*	161,6	*	1,4	ns	384,4	*	107,4	*	74,3	*	2,3	ns	181,4	*	3,0	ns	1,0	ns
Variety*Technology	9,5	*	327,3	*	24,2	*	0,3	ns	755,3	*	104,3	*	55,8	*	120,5	*	17,7	*	4,2	*	6,1	*

ns — not significant at p≤0.05.
* — significant at p≤0.05.

TDF—total dietary fiber, SDF—soluble dietary fiber, IDF—insoluble dietary fiber. WEAX, WUAX and TAX—water-extractable, water-unsoluble and total arabinoxylan content, respectively

Supplementary Table S4. Analysis of variance (two-way ANOVA). The effect of variety and technology of grain production on the content of phytates.

	IP3		IP4		IP5		IP6		Total phytates	
	F	p	F	p	F	p	F	p	F	p
Variety	0,1	ns	0,3	ns	1,6	ns	11,4	*	10,7	*
Technology	2,8	ns	2,7	ns	1,4	ns	0,0	ns	0,0	ns
Variety*Technology	2,7	ns	1,7	ns	3,5	ns	20,0	*	19,2	*

ns — not significant at p≤0.05.
* — significant at p≤0.05.

IP3-6 – inositol phosphates

Supplementary Table S5. Analysis of variance (two-way ANOVA). The effect of variety and technology of grain production on molecular properties of starch and water soluble arabinoxylan (WEAX).

	Starch Mw		Amylose Mw			Amylose content		WEAX Mw		FA content	
	F	p	F	p	p	F	p	F	p	F	p
Variety	86,2	*	14,3	*	*	3,2	ns	730,0	*	281,3	*
Technology	32,1	*	9,4	*	*	48,1	*	256,1	*	108,3	*
Variety*Technology	31,7	*	6,3	*	*	1,0	ns	734,5	*	33,6	*

ns — not significant at p≤0.05.
* — significant at p≤0.05.

Mw – molar mass, FA – ferulic acid content in AX molecules
